# League of VetaHumanz SuperPower Pack Program: Introducing Young People from Diverse Backgrounds to STEM Learning Activities and Veterinary Science Careers

**DOI:** 10.15695/jstem/v7i2.09

**Published:** 2024-02-01

**Authors:** Sandra F. San Miguel, Lindley McDavid

**Affiliations:** 1 College of Veterinary Medicine, Purdue University, West Lafayette, IN; 2 Evaluation and Learning Research Center, Purdue University, West Lafayette, IN

**Keywords:** Veterinary Medicine, STEM Engagement, Early Elementary, “Batman Effect”

## Abstract

League of VetaHumanz uses a nationwide network of university-community partnerships to provide veterinary STEM learning experiences for children who are more likely to lack access to enriching, supplemental educational opportunities due to systemic barriers based on their race, ethnicity, or socioeconomic status. To include participation beyond in-person programming, SuperPower Packs, self-guided, learning experiences, were developed. Leveraging social cognitive career theory and the “Batman Effect,” SuperPower Packs are designed to build self-efficacy, and seed STEM and veterinary science career aspirations by engaging children in STEM learning through connections with a veterinary role model. Four SuperPower Packs were developed. Beginning in the fall of 2021, for 17 months, 16,655 SuperPower Packs were distributed to children in 23 states. A small portion of children who received the game (3.8%, N = 614, 6-12 years old) returned evaluation surveys that measured activity engagement, likelihood of role model identification and demographics. Participants indicated variation in their experiences, but mean scale scores show desirable perceptions of engagement (M_Range_ = 2.38 – 2.90/3) and role model identification (M_Range_ = 2.15 – 2.94/3). These positive learning and role model experiences help set the stage to encourage youth to pursue similar learning and career opportunities in the future.

## INTRODUCTION

Since 2009, Purdue University College of Veterinary Medicine has led veterinary STEM programming for K-4 students with the goals of promoting healthy behaviors and diversifying the profession. All programs have been primarily supported by the Science Education Partnership Award (SEPA) program of the National Institute of General Medical Sciences (NIGMS) of the National Institutes of Health (NIH) with follow-on gift support. The League of VetaHumanz was launched on January 1, 2020, as an evolution of Purdue University College of Veterinary Medicine’s This is How We “Role” program (San Miguel et al., 2019). The League of VetaHumanz is an alliance of veterinary superheroes employed in academia, practice, research, government, and industry who are committed to engaging with under-resourced communities. League members serve as role models by providing access to veterinary STEM learning activities and positive social relationship support to under-resourced children (children who are more likely to have limited access to educational opportunities due to their race, ethnicity, or socioeconomic status), with the ultimate goal of encouraging young people’s interest, knowledge and pursuit of careers in the field.

Social cognitive career theory explains that contexts can support career development by fostering an individual’s self-efficacy or perception of ability to be successful in the given career or closely related tasks (Lent et al., 1994). Like its parent theory, social learning theory (Bandura, 1997), social cognitive career theory states that self-efficacy can be fostered through positive vicarious experiences (e.g., role models), verbal persuasion (e.g., internal and external encouragement), mastery experiences (e.g., successful task achievement) and physiological feedback (e.g., positive emotional states). Based on these foundational elements, League of VetaHumanz programming includes fun and engaging veterinary STEM learning experiences that enable children to experience success with challenging STEM veterinary content while having consistent supportive interactions with a diverse group of veterinary role models. Role models intentionally include racially and ethnically diverse veterinary professionals and veterinary medical students from a wide geographic area, representing the breadth of careers in the veterinary profession. Role models are committed to serving their community and pursuing opportunities to enact care and welcome to people from all backgrounds, especially young children from diverse and low socioeconomic status families. It is our hope that the children will see themselves, their families and their community in each role model. We also expect that positive social and learning experiences with caring role models will encourage youth to feel a sense of belonging (i.e., care, welcome, importance, and value; Ryan and Patrick, 2001) with each role model, and continue engaging in veterinary STEM and other related STEM learning contexts. Therefore, social cognitive career theory explains how the program, through role model interactions, learning success, and fun, can lay the groundwork for continued, long-term pursuit of experiences that support a career in the veterinary profession.

The League of VetaHumanz is the third evolution of over a decade of NIH SEPA-supported programming initiated and led by the College of Veterinary Medicine in collaboration with the Evaluation and Learning Research Center at Purdue University. The role model-delivered veterinary STEM lessons were piloted locally in 2012 through the Fat Dogs and Coughing Horses program (San Miguel et al., 2013), and then scaled nationally through the This Is How We “Role” program (San Miguel et al., 2019). Purdue University College of Veterinary Medicine continues to support and certify role model teams across the nation to deliver a 56-lesson veterinary STEM curriculum. Purdue program leaders assist teams in identifying community partners which are Title 1 schools or community organizations that are inclusive and place an emphasis on serving racially or ethnically minoritized children and/or children living at or near poverty. Role Model teams then collaborate with one or more partner schools or community organizations to deliver the veterinary curriculum as part of that organization’s programming. Role model teams are instructed to make consistent, weekly to monthly visits to build the supportive role model relationships that serve as the foundation for all program aims. In 2022 alone, role model teams made 102 total visits with 1,971 participations by children across 13 states.

Building on the success of these previous programs and a mission to reach more young people who did not have access to in-person programming, the SuperPower Pack program was launched in 2020. These self-guided learning activities were designed to leverage the growing network of university-community partnerships with the League of VetaHumanz and retain established elements of the traditional in-person delivery to broaden the delivery of quality, fun and enriching veterinary STEM educational activities to young people who are more likely to have limited access to supplemental learning opportunities.

### SuperPower Pack Program Foundation and Approach.

**Figure 1.**
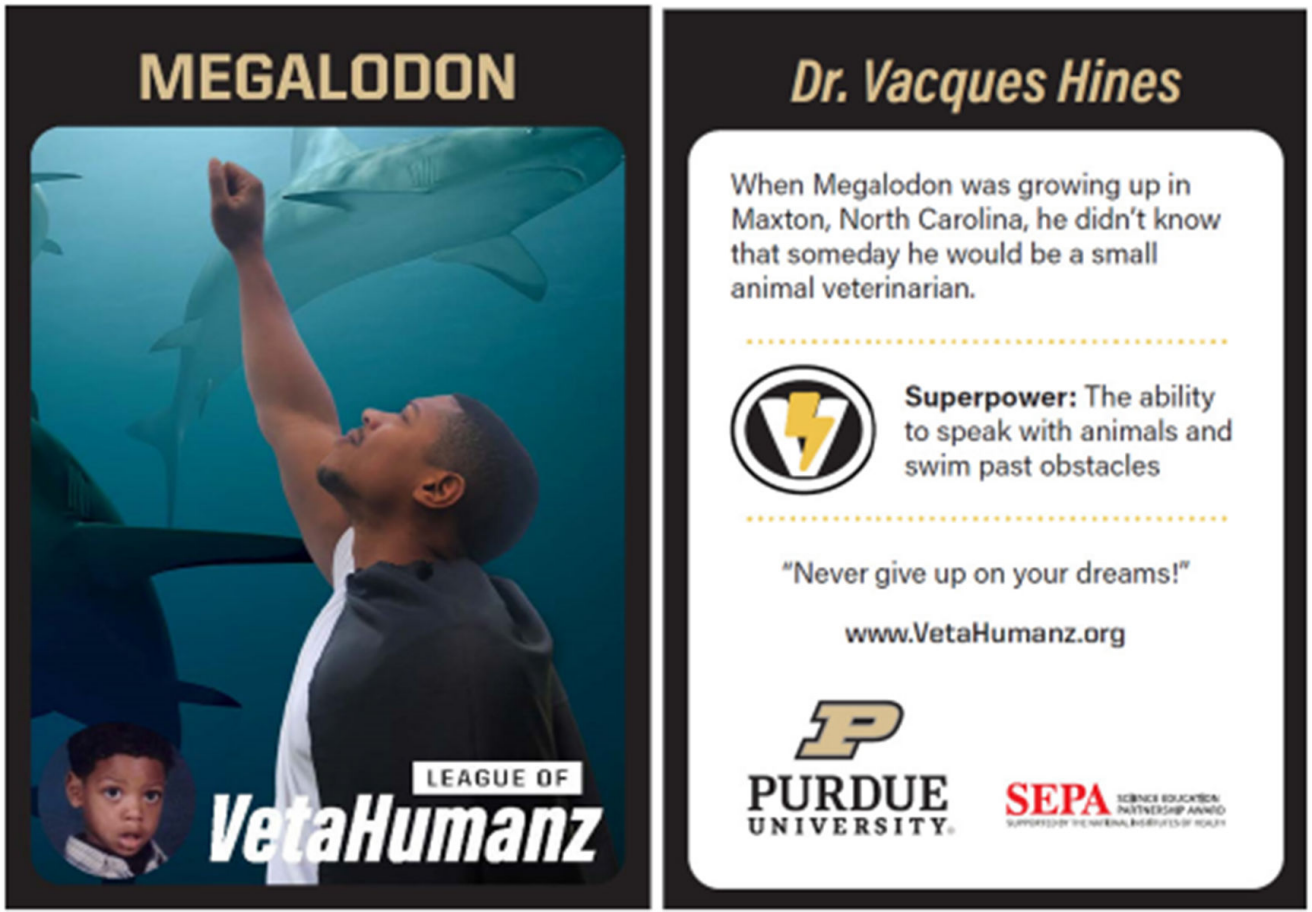
Veterinary superhero collectible card example. The front of each card provides images of the superhero as an adult and child. The back of the card provides supplementary information relatable to the reader.

As in the traditional in-person iterations of the program, the SuperPower Pack program remained premised on social cognitive career theory (Lent et al., 1994) and activity engagement (Carini et al., 2006; Fredricks et al., 2004). Each SuperPower Pack includes items designed to engage K-4 students in the veterinary profession by building self-efficacy, seeding career aspirations, and providing relatable role models. For example, each SuperPower Pack provides opportunities to build self-efficacy by promoting mastery experiences (e.g., learning human and animal anatomy) and positive emotional states (i.e., engaging in fun and lighthearted activities) coupled with the guidance and encouragement of a veterinary superhero role model (e.g., introduced with a collectible card and letter) (see Figure 1 for an example superhero collectible card). Each card and letter includes photographs of the veterinary superhero as an adult and child, provides background information about the superhero, their superpower, and an inspirational statement.

The activities are also designed to engage learners with interesting aspects of veterinary science and STEM through fun and lighthearted activities in order to help them persist on the task and increase their motivation to pursue similar learning experiences in the future (Carini et al., 2006; Fredricks et al., 2004). To help young people stay focused and interested in the activities, each SuperPower Pack is designed to engage youth behaviorally, cognitively and affectively. For example, children who are behaviorally engaged focus their behaviors to the activity itself and show limited off-task behaviors. Children who are cognitively engaged demonstrate interest in learning, following instructions and thoughtfulness in their participation. Children who are affectively engaged show positive emotional states and high arousal when completing the activity. In each activity, artwork is specifically designed to be joyful, represent people from diverse backgrounds and playfully depict characters and the learning activity. Activities also integrate age-appropriate humor, fun rhymes and movement all to help young people focus and support learning.

In tandem with the use of social cognitive career theory, SuperPower Packs also integrate what White et al. (2017) coined as the “Batman Effect”. In 2016, White and Carlson reported that children who completed a sorting task while pretending to be someone else (for example wearing a cape and pretending to be The Batman), demonstrated increased executive function, or focus on the task, compared to children working on the task as themselves. The researchers posited that taking on the persona of a competent alter ego improved children’s self-image and sense of competency, resulting in improved performance (White and Carlson, 2016). In 2017, White et al. reported that children who similarly psychologically self-distanced spent more time focused on completing a tedious task than taking breaks to play a video game, compared to children who did not pretend to be someone else. The researchers offered that pretending to be a competent other helped children put personal emotions/desires aside, as well as made the task more enjoyable, resulting in increased attention and time spent by the children on completing the task (White et al., 2017). In summary, this perspective suggests that children who are able to psychologically distance themselves from their own limitations in self-efficacy for a task demonstrate increased executive function and increased effort that results in improved task achievement (White and Carlson, 2016; White et al., 2017). To leverage the “Batman Effect” and increase task success, each SuperPower Pack contained superhero gear and a letter from the veterinary superhero role model encouraging the child to don the included gear and “borrow” their superpowers while performing the veterinary STEM activity.

## METHODS

### SuperPower Pack Developmental Criteria.

We established criteria for cost and packaging that would maximize distribution and facilitate use in contexts with minimal physical resources and adult supervision: a) SuperPower Packs would not require electricity or expensive electronic devices to use, b) the packaging would be of a size and weight that facilitated transport in a child’s backpack and minimized production and shipping costs, yet was substantial enough to allow individual shipping and protection from the elements, c) children would be able to perform activities with no or limited help from adults (e.g., children may need help with reading), and d) a postage paid, return addressed, evaluation survey would be included in each SuperPower Pack.

### SuperPower Pack Development and Content.

Led by the program director, a veterinarian and associate dean for engagement, who developed the SuperPower Pack approach and content, and monitored all program activities, the development team consisted of veterinarians and content specialists who served as content experts, translators, and featured role models; evaluation experts who developed tools for, and led, summative assessments; elementary school teachers/principals who aligned content with Next Generation Science Standards (NGSS Lead States, 2013); artists and graphic designers who made the materials appealing; program managers and administrative assistants who aided in activity development and distribution management; and a manufacturing company and a marketing agency who each provided economical and practical strategies for development and production of high quality physical materials. Each Super-Power Pack is described below. Further details, including aligned NGSS for individual SuperPacks are available on The League’s website (League of VetaHumanz, 2023).

#### Stat! SuperPower Pack.

**Figure 2.**
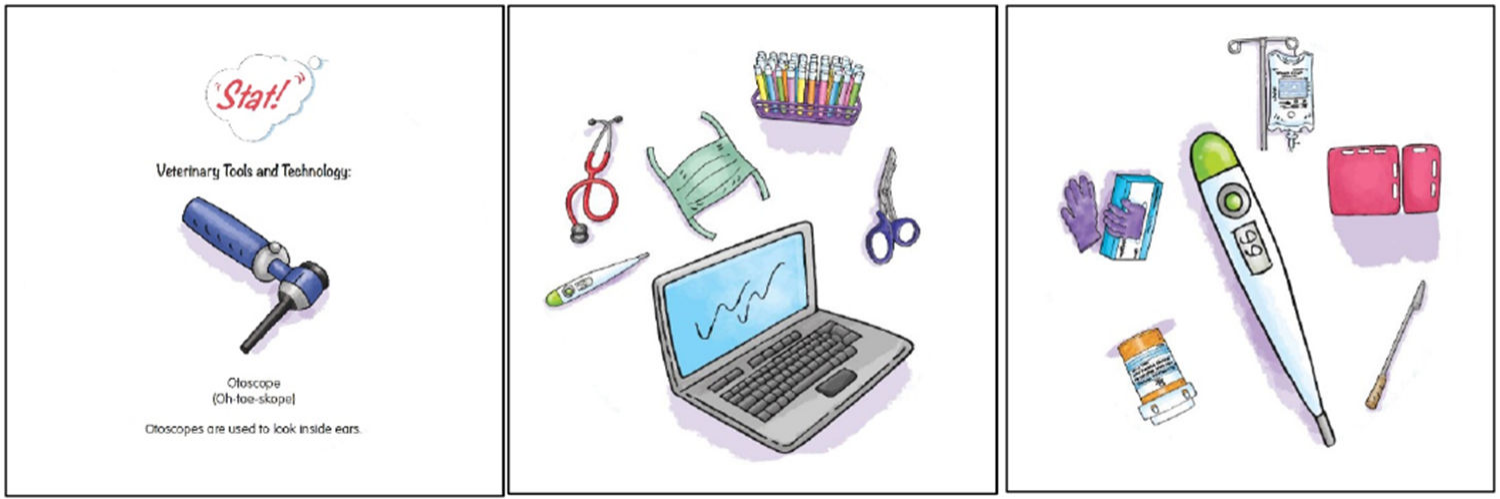
Stat! game card examples (learning card-left, playing cards-middle and right). In this example, the thermometer is the match.

The Stat! SuperPower Pack focuses on the items, tools, and instruments veterinarians use in emergencies. The game is designed for 2-4 players and for children over 5 years of age. The game includes learning cards and playing cards (Figure 2). Learning cards introduce each tool with an image, pronunciation of the name, and its purpose. Playing cards each show five images of instruments. One image is a match within any pair of cards. Players study the learning cards. Then players take turns revealing pairs of cards, and race to be the first one to identify the match. The game can be tailored to the skill level or developmental stage of the players. Beginner players can point to the match and say, “Stat!” Intermediate players can shout the name of the instrument followed by “Stat!” More advanced players can shout the name of the instrument followed by “Stat!,” and explain what the tool is used for to score the point. Stat! includes a superhero cape and features an emergency veterinarian superhero, Dr. Paula Johnson, AKA DreamCatcher. The postcard from DreamCatcher to the child reads:

Dear Superhero,

I’m a veterinarian who cares for sick or hurt animals in emergencies. I need to know what tools to use FAST! You can be an emergency vet, too! Use the yellow cards to learn about my tools. Then, grab two blue cards to see how fast you can find the same tool on each card- that’s the lifesaving tool. If you put on the cape while you play, you can be super speedy like me!

Use your powers for good!

DreamCatcher

#### Do You Have Diarrhea? SuperPower Pack.

**Figure 3.**
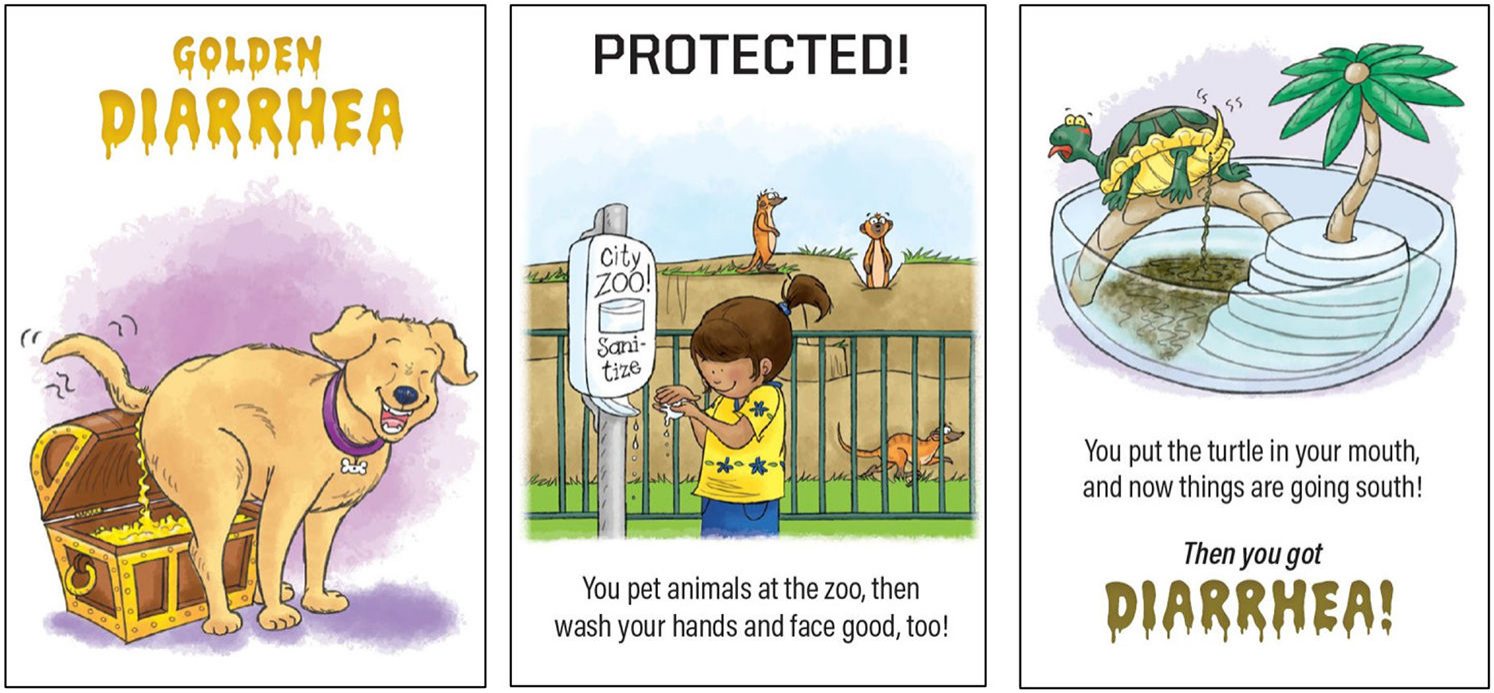
Do You Have Diarrhea? game card examples (golden diarrhea card-left, protection card–middle, diarrhea card-right).

The Do You Have Diarrhea? SuperPower Pack highlights the veterinarian’s role in public health. The game is designed for 2-5 players at least 6 years old. Players learn how people and other animals can get and prevent diarrhea, using the diarrhea cards and playing cards (Figure 3). Special cards, called Golden Diarrhea cards, are also included (Figure 3). Players try to collect pairs of diarrhea cards by asking other players if they have diarrhea. If the asked player has a diarrhea card, they must give it to the asker. However, if they have a protection card, they can keep their diarrhea card. The player who collects three Golden Diarrhea cards or has the most pairs of diarrhea cards wins.

Do You Have Diarrhea? includes a superhero cape and features a veterinarian superhero in academia, Dr. Sandra San Miguel, AKA Pink Phoenix. The postcard from Pink Phoenix to the child reads:

Dear Superhero,

I’m a veterinarian and teacher at Purdue University. I need to know all about diarrhea to help animals, even pigs and lizards, stay healthy. You can help animals, too! Collect pairs of diarrhea cards to learn how diarrhea spreads and use protection cards to stop it! If you put on the cape while you play, you can use my superpowers to stay healthy so you can save more animals!

Use your powers for good!

Pink Phoenix

#### Vaccines SuperPower Pack.

**Figure 4.**
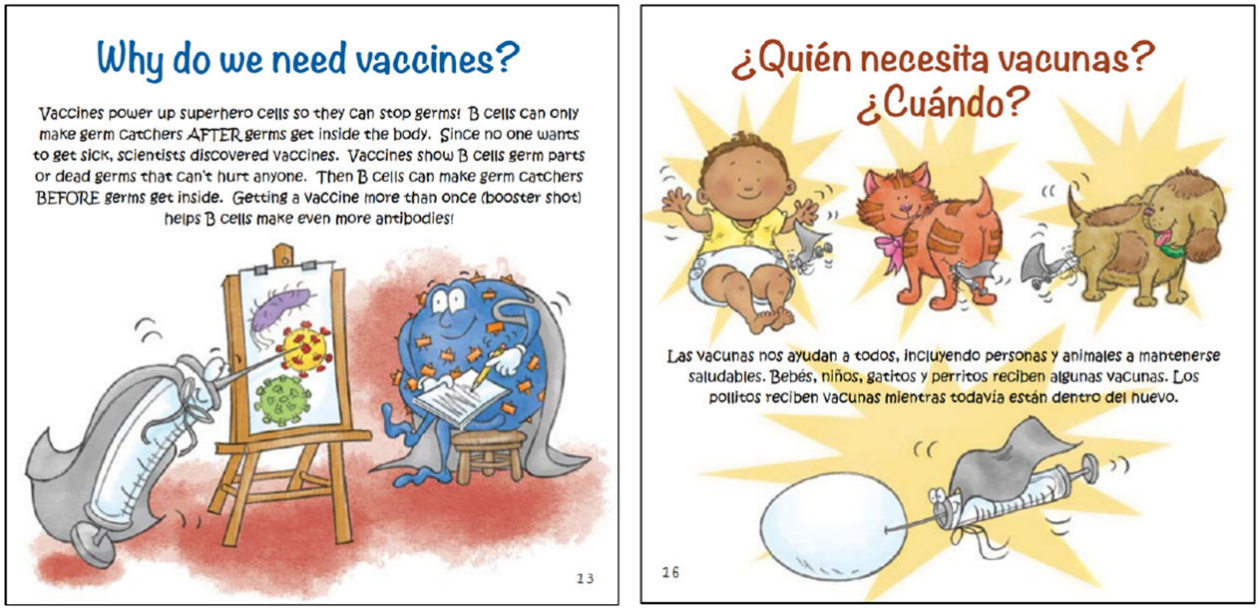
Sample pages from the book, VetaHumanz Need Vaccines, Too!*/¡VetaHumanz Necesitan Vacunas También!*

The Vaccines SuperPower Pack also emphasized the veterinarian’s role in public health by presenting immunology concepts. The book, VetaHumanz Need Vaccines, Too!/¡*VetaHumanz Necesitan Vacunas También!* is presented in both English and Spanish for children over 6 years of age. Children learn about how the immune system fights germs, as well as how vaccines are developed, tested, and used (Figure 4).

The Vaccines SuperPower Pack includes a superhero face mask with the imprint, “Power Up! Get Vaccinated!” and features four veterinarian superheroes in practice and academia, Dr. Harm HogenEsch AKA The Vaxinator, Dr. Allen L. Cannedy AKA Goat Vet, Dr. Tiffany Lyle AKA A New Dawn, and Dr. Suresh Mittal AKA Virus Fighter. The postcard to the child reads:

Dear Superhero,

I’m the veterinary scientist who hosts the VetaHumanz Live! podcast. I help people and animals stay healthy by sharing information from veterinary experts like myself. You can help people and animals stay healthy, too! Read the book and then listen to my podcast at www.VetaHumanz.org to learn about why vaccines are important. Then, put on the mask and use my superpowers to tell everyone to power up and get vaccinated!

Use your powers for good!

A New Dawn

#### Beast Moves SuperPower Pack.

The Beast Moves Super-Power Pack introduces how basic and applied sciences are used through comparative anatomy in veterinary medicine. The pack also introduces movement to encourage healthy habits related to physical activity. The game is designed for one or more players over 5 years of age.

**Figure 5.**
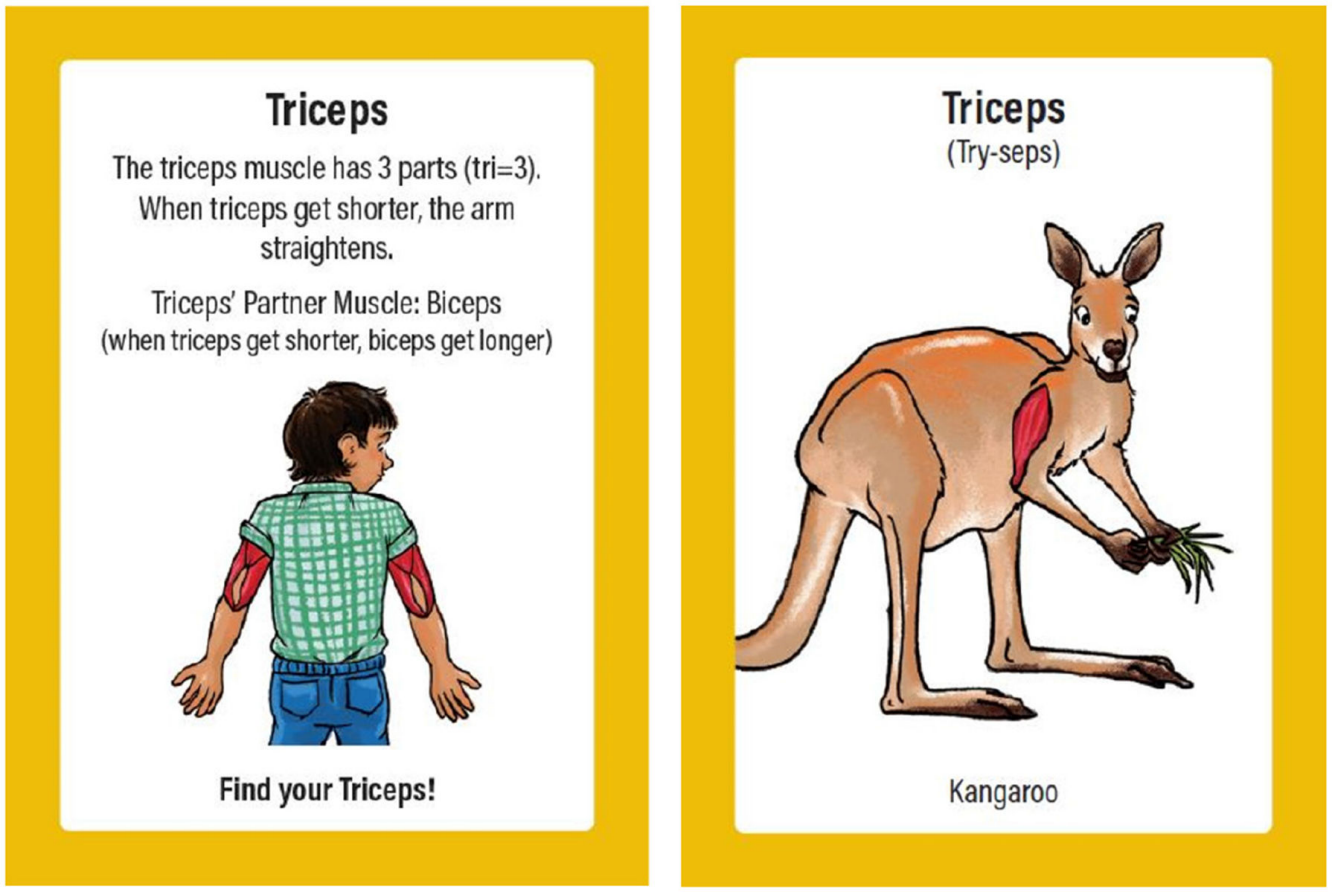
Beast Moves basic sciences card example.

**Figure 6.**
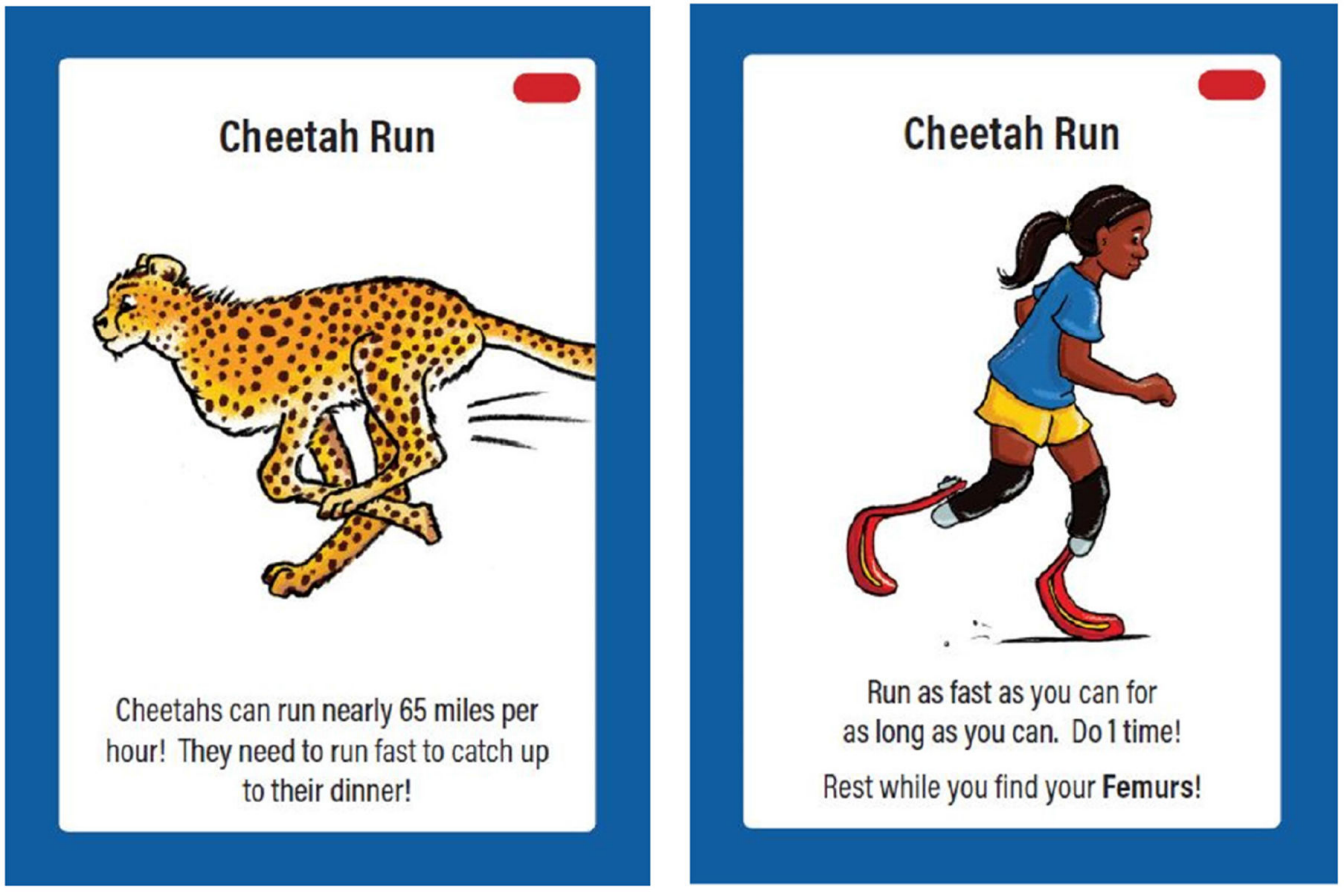
Beast Moves applied sciences card example.

The game includes basic sciences cards (Figure 5) and applied sciences cards (Figure 6). Basic sciences cards introduce a body part with images showing the location in a human and another animal, pronunciation of the name, and function. Applied sciences cards present an animal fact, provide players with a movement to perform, and ask players to identify one or more related body parts. Movements are identified according to difficulty level, and children are offered a variety of ways to play. A website and social media campaign served as companion resources to demonstrate movements.

Beast Moves includes a pair of tiger socks and features a small animal veterinarian superhero, Dr. Vacques Hines, AKA Megalodon. The postcard from Megalodon to the child reads:

Dear Superhero,

I’m a veterinarian who never gives up! I attack any obstacles in my way like a shark! I need to know all of the parts inside animals, and how they work, to keep animals healthy. I also need to bring my best every day-that means I have to stay healthy, too! You can be a vet like me! Use the yellow cards to learn about animal parts and the blue cards to practice your Beast Moves. If you put on the socks while you play, you can be smart and powerful like me!

Use your powers for good!

Megalodon

### SuperPower Pack Distribution.

Critical collaborations for distribution of SuperPower Packs included Purdue University’s Materials Management Distribution Center team who provided inventory management and shipping; role model teams and community partners who provided access to our target population; a digital communications team who made information about the program available to the public through websites and social media; and follow-on support from donors who enabled additional SuperPacks to be provided to children, beyond that supported by the NIH NIGMS SEPA grant. Follow-on support was especially needed and appreciated given the increase in supply costs due to the COVID-19 pandemic.

Briefly, each SuperPower Pack was batch manufactured with the total number of packs determined by the grant and sponsorship funds available. On arrival of the SuperPower Packs at Purdue University, all VetaHumanz teams were notified and given the opportunity to request packs to distribute to their in-person community partner(s) and other schools or organizations in their community that served under-resourced children. As well, Purdue University College of Veterinary Medicine’s in-person community partners, as well as other partners, including two school corporations, were notified and given the opportunity to request packs. Additionally, community entities serving under-resourced youth, who were not members of the Vetahumanz League, but heard about the program through the website or media releases, would request packs. The Purdue University team would process requests , and ship packs to the role model team leader or community organization leader, who then gave the SuperPower Packs to individual children. SuperPower Packs were distributed until the inventory was exhausted. The recipient of the requested SuperPower Packs was responsible for direct distribution to children to take home. Note that the Purdue team did not control if, where, or how the SuperPower Packs were utilized by children.

### Evaluation Procedures and Methods.

This research was determined to be exempt (Purdue University’s Institutional Review Board; Protocol #2021 1136, 2020-1762). Four SuperPower Packs were produced, Stat! Do You Have Diarrhea?, Vaccines, and Beast Moves! In total, 16,654 SuperPower Packs were shipped to children in 23 states. A companion website for each SuperPower Pack provided additional information, including videos of unboxings and play, as well as applicable academic standards (https://vet.purdue.edu/vetahumanz/super-power-packs/).

Each SuperPower Pack contained a return postage paid evaluation survey consisting of either a single sheet of paper that could be folded, taped, and returned or a single sheet of paper with a peel and seal envelope. The survey was kept short to reduce the burden of survey completion and return by children and their families. The evaluation included measures to assess the foundational principles of the program including opportunities for vicarious experiences and verbal persuasion (i.e., role model identification and the “Batman Effect”), and positive mastery and physiological experiences (i.e., engagement).

#### Opportunity for Vicarious Experiences and Verbal Persuasion.

Three items were developed to assess the potential for role model identification based on the motivational theory of role modeling (Morgenroth et al., 2015). Specifically, the questions assessed children’s perceptions that they are like and can do things like the role model (*I can be like the superhero*), that the superhero presents a desirable behavior (*The superhero is successful in what they do*) and that they want to be like the superhero (*I want to be like the superhero*). Youth responded on a three-point scale of “No!”, “I don’t know!” or “Yes!”. Items demonstrated marginal internal consistency in this sample (α = .56). As the scale performance was limited by the inclusion of only three items and response choices to accommodate the delivery mode of the survey, and item responses were significantly and positively correlated with the scale sum score (*r* = .48-.82), the scale was used as designed.

Two items were used to assess embodiment of the role model or the “Batman Effect” (White and Carlson, 2016; White et al., 2017) in each SuperPower Pack. Youth responded to questions that assessed their use of the superhero gear (*Did you wear* [superhero gear] *while playing?*) and if they imagined they were the superhero (*Did you pretend to be the veterinarian superhero while you played?*). The Vaccines SuperPower Pack contained a superhero mask with a book instead of a game as the STEM activity and questions were adjusted accordingly. Youth responded to one item that indicated if they would enact a role model behavior regarding vaccine promotion as depicted in the book (*Will you act like the superhero and tell people to get vaccinated?*).

#### Positive Mastery and Physiological Experiences.

The abbreviated version of the Engagement in Science Learning Activities Scale (Chung et al., 2016) was used as a proxy of student positive mastery and physiological experiences. Engagement captures the degree that learning contexts promote emotional (“*When I played the game, I felt happy*.” “*When I played the game, I felt bored*.”), cognitive (“*When I played the game, time went by quickly*.”) and behavioral (“*When I played the game, I paid attention to the game*.”) states and predicts motivation to learn and learning. The scale has been used in previous iterations of the program and demonstrated adequate reliability and internal consistency in similar samples (San Miguel et al., 2019) and was modified when needed to refer to the presented SuperPower Pack activity.

#### Demographic Information.

Participants’ age, gender identity, race and ethnicity were collected.

#### Fidelity of SuperPower Pack Activity Use.

Two items were developed to ensure that only those who used the SuperPower Pack before completing the survey would be included in the study sample. The first question asked about their activity dosage (*How many times did you play the game?*) and the second question asked if they could identify the introduced superhero (*Who was the veterinarian superhero for this game?*).

Where appropriate, scale internal consistencies, and descriptive statistics including the mean, standard deviation and range of each scale or item were calculated for the psychosocial variables. Frequencies and ranges were calculated for all demographic variables. All analyses were executed in SPSS version 25 (IBM, 2017).

## RESULTS

The Stat! SuperPower Pack was distributed to 3,127 children with a 4.4% (138) survey response rate (Table 1). The Do You Have Diarrhea? SuperPower Pack was distributed to 4,602 children with a 3.19% (147) survey response rate (Table 2). The Vaccines SuperPower Pack was distributed to 5,996 children with a 3.5% (210) survey response rate (Table 3). The Beast Moves SuperPower Pack was distributed to 2,930 children with a 4.1% (119) survey response rate (Table 4). Youth were from 6-12 years old, nearly half girls and boys, and were from diverse racial and ethnic backgrounds with 62-82% of participants being from non-white or more than one racial and ethnic background.

Across all SuperPower Packs most children indicated that they played the activity one time (36-64%) or three times (20-42%) and were able to indicate the veterinarian superhero correctly (81-93%). However, for the Do You Have Diarrhea? SuperPower Pack only 52% of participants correctly indicated the veterinary superhero. Participants had moderate to strong and positive perceptions of SuperPower Pack engagement with consistently higher perceptions that they “can be like the superhero” and “the superhero is successful” (response range = 2.37 - 2.94) compared to that they “want to be like the superhero” (range response = 2.15-2.38/3). Most children also wore the cape or socks (63-94%) when playing or indicated that they would act like the superhero (75%). Last, children also had strong and positive perceptions of their cognitive (2.52-2.86), emotional (2.58-2.90) and behavioral (2.44-2.83) engagement across all SuperPower Packs.

## DISCUSSION

Based on the degree of use, role model identification, role model embodiment and engagement, and the diversity in race and ethnicities of children reached, the SuperPower Pack Program was an effective strategy to introduce diverse children to relatable veterinary role models, and deliver an engaging veterinary STEM curriculum, apart from in-person programming.

The League of VetaHumanz uses veterinarian and veterinary medical student role models to deliver our STEM curriculum in person. As social cognitive career theory posits (Lent et al., 1994), positive role models are essential to develop desirable career impressions and aspirations. Furthering the visibility of relatable role models is also critical for recruiting a diverse group of students to the veterinary profession, as the dearth of racial and ethnic diversity in the veterinary profession can turn even our youngest students away and perpetuate the lack of workforce diversity. Eccles (2011) theorized that individuals do not pursue certain career options because of lack of awareness, inaccurate information, or lack of feelings that success is attainable in that occupation. For a stereotypical white profession, providing under-resourced children access to under-represented veterinary role models is critical. Dasgupta (2011) refers to experts and peers in a specific field as “social vaccines” because these role models enhance career aspirants’ views of social belonging in that occupation. The sense of belonging reduces occupational stereotypes and self-doubt that success is not viable in that career. Dasgupta (2011) further theorizes that access to relatable role models enhances positive attitudes toward a profession such that individuals, especially minorities, will identify with and be motivated to pursue that profession.

The SuperPower Pack program was challenged in that in-person role models could not be used. We selected collectible cards and a get-to-know-you letter as a replacement for live role models based on work by Gibson (2004), who defined role models as cognitive constructions that individuals create from multiple others who are similar to themselves and who have attributes and behaviors that the individual desires to emulate or avoid. In lieu of in-person role models, we provided children the materials for which to create their own cognitive constructions of relatable role models that incorporated the key features from the Motivational Theory of Role Modeling (Morgenroth et al., 2015): 1) a photograph of the role model as an adult and child to facilitate the child envisioning themselves as a veterinarian, 2) the story of where the role model grew up, their dreams, and their current career to demonstrate goal attainment was possible, 3) the role models’ superpowers to reflect behaviors that help them be successful, and 4) an inspirational message to provide encouragement for the child. The results of this study demonstrate the effectiveness of this strategy and support the view that role models can have profound effects even without a physical presence (Gibson 2004; Lockwood and Kunda, 1997).

We selected game play as the mode to deliver veterinary STEM educational materials to increase young people’s interest and knowledge in veterinary STEM content and careers (for all but the Vaccines SuperPower Pack). Game play reinforces verbal and nonverbal communication skills, learning, and critical thinking, and helps children gain confidence through developing multiple approaches to overcoming obstacles, as they interact with their peers (Becker et al., 2009). Game play fosters social interaction where children can collaborate, accept, and provide feedback, display leadership, appreciate alternative perspectives, develop interpersonal skills, and explore ethical choices (Becker et al., 2009). Game play is also an effective strategy for empowering children during self-directed inquiry (Becker et al., 2009). The activities were also designed to avoid the need for personal electronic devices as our target population of under-resourced children may not have access to this resource.

To extend the time children were engaged in the SuperPower Pack STEM activities, and assist children in visualizing themselves as veterinarians, we used the “Batman Effect.” White and Carlson (2016) theorize that role-play allows children to form a more competent self-image resulting in perseverance and improved performance. SuperPower Packs leveraged the “Batman Effect,” by including collectible cards to introduce veterinary role models and careers within the veterinary profession, and personal notes from the veterinary superhero to encourage the child to take on their persona of a veterinary superhero while playing by donning the enclosed superhero gear. Although the contribution to the results directly attributed to the “Batman Effect” could not be directly measured through this study, many children reported engaging with the STEM activities multiple times and pretended to be the superhero veterinarian and use their veterinary superhero powers while playing. Additionally, our team directly observed and received reports of children wearing their VetaHumanz capes in the community, or wearing their pants rolled up in school to show off their Beast Moves socks. It is our hope that these behaviors indicate that children are interested in and proud of their program involvement.

One major limitation of this program evaluation was the low survey return rate. To improve participation, we attempted various iterations of the evaluation survey design and return process across the SuperPower Pack distribution with little success. The Stat! and Do You Have Diarrhea? surveys were a single sheet of paper that needed to be folded, taped, and returned. These SuperPower Packs had the highest (4.4%) and lowest (3.2%) return rates, respectively. The Vaccines SuperPower Pack surveys contained the survey on a piece of paper with a peel and seal envelope for return and had a similar return rate (3.5%). We printed “Please be a SUPERHERO and Return your survey in this envelope! It’s free!” on the The Beast Moves SuperPower Pack envelope and had our second highest return rate (4.1%). The next iterations of production will include an envelope with an encouraging message, and the survey will also be printed on brightly colored paper and include a peel and seal envelope.

Lastly, the process of developing and distributing SuperPower Packs offered many challenges given the large number of people and collaborations required to design, produce and distribute the activities. Stalled production as a result of the pandemic, included paper and cardboard shortages and transportation issues resulting in substitutions, increased costs of production, and delays. Examples of challenges in distribution included a refusal of a shipment by a school principal because the pallets of SuperPower Packs had been labeled, “Purdue University College of Veterinary Medicine Diarrhea Kits,” and a superintendent of a school district who blocked distribution of the Vaccines SuperPower Packs due to the volitile political context surrounding vaccines and vaccination, after the requesting school had received the shipment. Engaging all partners at the planning stage, appreciating and utilizing each other’s strengths and expertise, and engaging in extensive, regular, open communications made navigating challenges possible. Despite all of these setbacks, our results demonstrate that through leveraging the role model team and community partner relationships within, and the extensive reach of, The League of VetaHumanz network, we were able to successfully distribute SuperPower Packs to a diverse group of children (62-82% of children reported that they were from non-white backgrounds) in 23 states, an integral part of our mission to introduce under-resourced children to the veterinary profession through STEM activities.

In addition to our study, the SuperPower Pack program was externally reviewed and recognized. All of the SuperPower Packs, except Stat!, were submitted to Academics’ Choice Awards^™^ for external review and consideration for Brain Toy awards. Submissions are reviewed by a panel of judges and independently evaluated for development of general and higher order thinking skills, addressing academic standards, character building, engagement, ease of use, quality/durability, and innovation (Academics’ Choice Awards, 2022). Each of the submitted SuperPower packs, Do You Have Diarrhea?, Vaccines, and Beast Moves! were recipients of 2022 Academics’ Choice Brain Toy Awards. Additionally, the SuperPower Pack program was recognized with a 2022 Inspiring Programs In STEM Award from INSIGHT Into Diversity magazine, the foremost diversity, equity, and inclusion publication in academia. Inspiring Programs in STEM Award winners are evaluated by INSIGHT Into Diversity magazine based on efforts to inspire and encourage diverse, young people to consider careers in STEM (Insight Staff, 2022).

Despite limitations and challenges, the evidence that we present supports that through The League of VetaHumanz SuperPower Pack program, children from diverse backgrounds and ages were able to receive and use the SuperPower Packs, and even with low response rates, consistently report positive levels of engagement and positive perceptions of the role models. Overall, these findings set the stage for innovative ways to motivate young people to pursue new, similar learning opportunities and related career pathways in the future.

## Figures and Tables

**Table 1.** Stat! Summary of Results.

Theoretical Foundation	Program Element	Survey Question	*Mean (Range)* or Frequency
**Fidelity of SuperPower Pack activity use**	Initial activity use	*How many times did you play the game?*	Once, 64%
			Twice, 17%
			Thrice, 20%
	Awareness of role model	*Who was the veterinarian superhero for this game?*	81% Correct*
**Opportunity for vicarious experiences and verbal persuasion**	Potential for role model identification	*I can be like the superhero*	2.67 (1-3)
		*The superhero is successful in what they do*	2.94 (1-3)
		*I want to be like the superhero*	2.37 (1-3)
**Role model embodiment**	“Batman Effect”	*Did you wear cape while playing?*	80% Yes
		*Did you pretend to be the veterinarian superhero while you played?*	63% Yes
**Positive mastery and physiological experiences**	Activity engagement	Emotional engagement: *When I played the game, I felt happy. When I played the game, I felt bored (reversed scored)*	2.90 (1-3)
		Cognitive engagement: *When I played the game, time went by quickly*	2.81 (1-3)
		Behavioral engagement: *When I played the game, I paid attention to the game*	2.89 (1-3)
**Participant Demographics**	Age	9.04 (6-12)	
	*Gender*	55% girls, 43% boys, < 1% additional	
	*Race/Ethnicity*	19% Black, 9% Latinx, 42% More than 1, 1% Asian/Native American, 36% White, 4% Additional	

Note: * Some participants listed their own names, versus the activity’s role model, as the veterinarian superhero for this game. These responses were excluded from this question.

**Table 2.** Do You Have Diarrhea? Summary of Results.

Theoretical Foundation	Program Element	Survey Question	*Mean (Range)* or Frequency
**Fidelity of SuperPower Pack activity use**	Initial activity use	*How many times did you play the game?*	Once, 45%
			Twice, 16%
			Thrice, 39%
	Awareness of role model	*Who was the veterinarian superhero for this game?*	52% Correct*
**Opportunity for vicarious experiences and verbal persuasion**	Potential for role model identification	*I can be like the superhero*	2.61 (1-3)
		*The superhero is successful in what they do*	2.76 (1-3)
		*I want to be like the superhero*	2.15 (1-3)
**Role model embodiment**	“Batman Effect”	*Did you wear cape while playing?*	94% Yes
		*Did you pretend to be the veterinarian superhero while you played?*	53% Yes
**Positive mastery and physiological experiences**	Activity engagement	Emotional engagement: *When I played the game, I felt happy. When I played the game, I felt bored (reversed scored)*	2.81 (1-3)
		Cognitive engagement: *When I played the game, time went by quickly*	2.61 (1-3)
		Behavioral engagement: *When I played the game, I paid attention to the game*	2.83 (1-3)
**Participant Demographics**	Age	8.84 (6-12)	
	*Gender*	42% girls, 54% boys, 1% additional	
	*Race/Ethnicity*	30% Black, 16% Latinx, 11% More than 1, 2% Asian/Native American, 38% White, 2% Additional	

Note: *Some participants listed their own names, versus the activity’s role model, as the veterinarian superhero for this game. These responses were excluded from this question.

**Table 3.** Vaccines Summary of Results.

Theoretical Foundation	Program Element	Survey Question	*Mean (Range)* or Frequency
**Fidelity of SuperPower Pack activity use**	Initial activity use	*How many times did you play the game?*	Once, 64%
			Twice, 14%
			Thrice, 21%
	Awareness of role model*	*Who was the veterinarian superhero for this game?*	84% Correct
**Opportunity for vicarious experiences and verbal persuasion**	Potential for role model identification	*I can be like the superhero*	2.67 (1-3)
		*The superhero is successful in what they do*	2.83 (1-3)
		*I want to be like the superhero*	2.20 (1-3)
**Role model embodiment**	“Batman Effect”	*Will you act like the superhero and tell people to get vaccinated?*	5% No
			20% I don’t know
			75% Yes
**Positive mastery and physiological experiences**	Activity engagement	Emotional engagement: *When I played the game, I felt happy. When I played the game, I felt bored (reversed scored)*	2.58 (1-3)
		Cognitive engagement: *When I played the game, time went by quickly*	2.86 (1-3)
		Behavioral engagement: *When I played the game, I paid attention to the game*	2.44 (1-3)
**Participant Demographics**	Age	8.74 (6-12)	
	*Gender*	49% girls, 49% boys, < 0.5% additional	
	*Race/Ethnicity*	20% Black, 9% Latinx, 33% More than 1, 2% Asian/Native American, 33% White, 3% Additional	

Note: *Some participants listed their own names, versus the activity’s role model, as the veterinarian superhero for this game. These responses were excluded from this question.

**Table 4.** Beast Moves Summary of Results.

Theoretical Foundation	Program Element	Survey Question	*Mean (Range)* or Frequency
**Fidelity of SuperPower Pack activity use**	Initial activity use	*How many times did you play the game?*	Once, 36%
			Twice, 22%
			Thrice, 42%
	Awareness of role model	*Who was the veterinarian superhero for this game?*	93% Correct*
**Opportunity for vicarious experiences and verbal persuasion**	Potential for role model identification	*I can be like the superhero*	2.56 (1-3)
		*The superhero is successful in what they do*	2.75 (1-3)
		*I want to be like the superhero*	2.38 (1-3)
**Role model embodiment**	“Batman Effect”	*Did you wear cape while playing?*	63% Yes
		*Did you pretend to be the veterinarian superhero while you played?*	53% Yes
**Positive mastery and physiological experiences**	Activity engagement	Emotional engagement: *When I played the game, I felt happy. When I played the game, I felt bored (reversed scored)*	2.67 (1-3)
		Cognitive engagement: *When I played the game, time went by quickly*	2.52 (1-3)
		Behavioral engagement: *When I played the game, I paid attention to the game*	2.78 (1-3)
**Participant Demographics**	Age	7.55 (6-12)	
	*Gender*	52% girls, 48% boys, < 1% additional	
	*Race/Ethnicity*	39% Black, 27% Latinx, 12% More than 1, 3% Asian/Native American, 18% White, 1% Additional	

Note: *Some participants listed their own names, versus the activity’s role model, as the veterinarian superhero for this game. These responses were excluded from this question.

## ABBREVIATIONS

NGSS: Next Generation Science Standards
NIGMS: National Institute of General Medical Sciences
NIH: National Institutes of Health
SEPA: Science Education Partnership Award
